# Subtype-specific regulation of P2X3 and P2X2/3 receptors by phosphoinositides in peripheral nociceptors

**DOI:** 10.1186/1744-8069-5-47

**Published:** 2009-08-11

**Authors:** Gary Mo, Louis-Philippe Bernier, Qi Zhao, Anne-Julie Chabot-Doré, Ariel R Ase, Diomedes Logothetis, Chang-Qing Cao, Philippe Séguéla

**Affiliations:** 1 Montreal Neurological Institute, Department of Neurology & Neurosurgery, McGill University, Montreal, Canada; 2 Department of Structural and Chemical Biology, Mount Sinai School of Medicine, New York, USA; 3 Department of Bioscience, AstraZeneca R&D Montreal, Montreal, Canada

## Abstract

**Background:**

P2X3 and P2X2/3 purinergic receptor-channels, expressed in primary sensory neurons that mediate nociception, have been implicated in neuropathic and inflammatory pain responses. The phospholipids phosphatidylinositol 4,5-bisphosphate (PIP_2_) and phosphatidylinositol 3,4,5-trisphosphate (PIP_3_) are involved in functional modulation of several types of ion channels. We report here evidence that these phospholipids are able to modulate the function of homomeric P2X3 and heteromeric P2X2/3 purinoceptors expressed in dorsal root ganglion (DRG) nociceptors and in heterologous expression systems.

**Results:**

In dissociated rat DRG neurons, incubation with the PI3K/PI4K inhibitor wortmannin at 35 μM induced a dramatic decrease in the amplitude of ATP- or α,β-meATP-evoked P2X3 currents, while incubation with 100 nM wortmannin (selective PI3K inhibition) produced no significant effect. Intracellular application of PIP_2 _was able to fully reverse the inhibition of P2X3 currents induced by wortmannin. In *Xenopus *oocytes and in HEK293 cells expressing recombinant P2X3, 35 μM wortmannin incubation induced a significant decrease in the rate of receptor recovery. Native and recombinant P2X2/3 receptor-mediated currents were inhibited by incubation with wortmannin both at 35 μM and 100 nM. The decrease of P2X2/3 current amplitude induced by wortmannin could be partially reversed by application of PIP_2 _or PIP_3_, indicating a sensitivity to both phosphoinositides in DRG neurons and *Xenopus *oocytes. Using a lipid binding assay, we demonstrate that the C-terminus of the P2X2 subunit binds directly to PIP_2_, PIP_3 _and other phosphoinositides. In contrast, no direct binding was detected between the C-terminus of P2X3 subunit and phosphoinositides.

**Conclusion:**

Our findings indicate a functional regulation of homomeric P2X3 and heteromeric P2X2/3 ATP receptors by phosphoinositides in the plasma membrane of DRG nociceptors, based on subtype-specific mechanisms of direct and indirect lipid sensing.

## Background

P2X receptors are nonselective cation channels gated by ATP and expressed on a variety of cell types in mammals, including neurons, glia, epithelial cells and smooth muscle cells [1,2]. In mammals, seven subunits (P2X1-7, new NC-IUPHAR nomenclature) have been identified, which associate to form homo- and heterotrimeric receptor-channels [3-6]. Extensive evidence indicates that P2X receptors are involved in both peripheral and spinal pain transmission [7-9]. Particularly, the expression of the P2X3 subtype is selective for the non-peptidergic small-diameter dorsal root ganglion (DRG) neurons, which are associated with nociceptive transmission [10-12]. *In vivo *studies have provided evidence that the activation of homomeric P2X3 and heteromeric P2X2/3 ATP receptors contributes to acute nociceptive behavior, hyperalgesia and allodynia [13-15].

Much attention has been brought to phosphoinositides as major signaling molecules at plasma membranes. The precursor of all phosphoinositides, phosphatidylinositol (PI), is the most abundant, followed by PI(4,5)P_2 _(PIP_2_) and PI4P, each comprising 1% of the total phospholipids in the plasma membrane. PI(3,4,5)P_3 _(PIP_3_) is considerably less abundant. These phospholipids are produced by selective lipid kinases, namely phosphoinositol 3-kinase (PI3K), phosphoinositol 4-kinase (PI4K) and phosphoinositol 5-kinase, which phosphorylate the inositol ring at the 3', 4' or 5' positions. Synthesis of PIP_2 _is completed through successive phosphorylations by PI4K and phosphoinositol(4) 5-kinase. Formation of PIP_3 _from PIP_2 _requires additional phosphorylation by PI3K. Phosphoinositides exert their regulatory role either indirectly, for example as precursors of the phospholipase C-generated second messengers inositol 1,4,5-trisphosphate and diacylglycerol, or directly by interacting with membrane proteins via electrostatic binding to positively charged residues, thus controlling their subcellular localization and/or their activity [16,17].

Recent studies have revealed an increasing number of ion channels interacting with phosphoinositides, including Kir [18], TRP [19], P2X [20], NMDA [21] and BK [22]. Following the work of Fujiwara and Kubo [23] on the modulation of P2X2 receptors by phosphoinositides, other P2X receptors were found to be sensitive to phospholipids [24-26]. The present study explored the functional interaction of PIP_2 _and PIP_3 _with native and heterologously expressed P2X3 and P2X2/3 receptor-channels and its physiological consequences. Using isolated DRG neurons in culture and patch-clamp recording, we report a strong modulation of homomeric P2X3- and heteromeric P2X2/3-mediated responses by wortmannin-induced phosphoinositide depletion. By means of two-electrode voltage-clamp and inside-out patch recordings in the *Xenopus laevis *oocyte expression system, we also provide functional evidence that PIP_2 _and PIP_3 _modulate the speed of recovery of P2X3 and P2X2/3 receptor channels. Finally, using an *in vitro *lipid binding assay we show that the binding of phospholipids to P2X3 subunit is likely indirect. The proximal C-terminal region of P2X2 subunit directly interacts with several anionic phospholipids, including PIP_2_, and thus confers direct phospholipid modulation to the heteromeric P2X2/3 receptor channels.

## Methods

### Cell Culturing and mutagenesis

DRGs from lumbar segments were extracted from Sprague-Dawley rats age 4–6 weeks (Charles River Canada) under deep anaesthesia induced by halothane (Sigma-Aldrich). Extracted DRGs were placed in ice-cold oxygenated DMEM (Gibco) for removal of connected tissue and dura matter. The isolated DRGs were then placed into DMEM containing 1 mg/mL of papain and 2 mg/mL collagenase type II (both from Sigma Aldrich) and incubated for 1 h at 37°C. After enzymatic digestion, the DRGs were transferred into DMEM containing 10% FBS and 1% L-glutamine, dissociated into single neurons by means of trituration using fire-polished pipettes. Dissociated neurons were then plated onto 35 mm cell culture dishes (Sarstedt) coated with laminin (BD Bioscience) and poly-D-lysine (Sigma-Aldrich), and cultured for 48 h at 37°C and 100% humidity in F-12 media (Gibco) containing 10% FBS, 1% L-glutamine and 100 U/mL penicillin and streptomycin. The culturing media was also supplemented with 30 ng/mL of NGF (Sigma-Aldrich). Culturing media for the HEK293 cells was DMEM supplemented with 10% fetal bovine serum, 1% penicillin and streptomycin, 1% MEM non-essential amino acids, and 1% glutamine (all from Gibco). HEK293 cells were transiently cotransfected with EGFP and wild-type or mutant rat P2X3 in pcDNA3 using PolyFect Transfection Reagent (Qiagen) according to the manufacturer's instructions. Residues K348, K354, R356, K357, and R367 of P2X3 were mutated into glutamine using Quikchange (Stratagene) site-directed mutagenesis and mutations were confirmed by sequencing.

### Patch-clamp recordings in DRG neurons and transfected HEK293 cells

Whole-cell patch-clamp recordings (V_h _of -60 mV) on DRG neurons were conducted using pipettes filled with internal solution, pH 7.2, containing (in mM): 130 K-gluconate, 1 MgCl_2_, 5 EGTA, 10 HEPES, 3 MgATP, and 0.4 GTP. Drug applications were performed using a fast microperfusion system at a rate of 1 mL/min (SF-77B, Warner Instruments, Morris Plains, NJ). The standard perfusion solution, pH 7.4, comprised (in mM): 152 NaCl, 5 KCl, 2 CaCl_2_, 1 MgCl_2_, 10 HEPES, and 10 glucose. HEK293 cells were used for electrophysiological recordings (V_h _of -60 mV) 24–48 h after transfection. The pipette solution contained (in mM): 120 K-gluconate, 1 MgCl_2_, 4 NaOH, and 10 HEPES (pH 7.2). The perfusion solution, pH 7.4, comprised (in mM): 140 NaCl, 5 KCl, 2 CaCl_2_, 2 MgCl_2_, 10 HEPES, and 10 glucose. Membrane currents were recorded using an Axopatch 200B amplifier, digitized with a Digidata 3200A interface (Axon Instruments, Foster City, CA.), and acquired at a frequency of 2 kHz using pClamp 9. Osmolarity of external solutions were adjusted to 300 mOsm and that of pipette solutions to 280 mOsm. All experiments were carried out at room temperature (20–23°C). Recording electrodes were produced by pulling borosilicate glass tubes using a P-97 puller (both from Sutter Instrument, Novato, CA.), and fire-polishing with a MP-830 microforge (Narishige, Tokyo, Japan) to a tip resistance of 3–6 MΩ when filled with ICS. The culturing chambers were also used as the recording chamber. Results were expressed as amplitude of peak currents evoked by α,β-meATP or as current density, defined as the ratio of peak amplitude over membrane capacitance (pA/pF). For measuring recovery, the amplitude of the third P2X3 response was compared to the first and expressed as a percentage (rundown ratio). The results obtained after 2 h drug incubation were always compared to those obtained after 2 h incubation in culturing media containing DMSO (vehicle). Membrane capacitance (C_m_) and series resistance (R_s_) were measured through the peak amplitude and decay constant of transients induced by repetitive depolarizing pulses of 10 mV.

### Voltage-clamp and macropatch recordings in Xenopus oocytes

Oocytes were surgically removed from Tricaine-anesthetized female *Xenopus laevis *frogs and were incubated in OR2 solution containing 1–2 mg/ml type IA collagenase at room temperature for 2 h under agitation. Stage V and VI oocytes were then manually defolliculated before nuclear or cytoplasmic microinjection of plasmid DNA coding for P2X2 (5 ng) and tandem P2X2/3 (5 ng) or mRNA coding for P2X3 (40 ng). After injection, oocytes were incubated in Barth's solution containing 1.8 mM CaCl_2 _at 19°C for 24 to 48 h before electrophysiological recordings. Two-electrode voltage-clamp recordings (V_h _= -60 mV) were performed using glass pipettes (1–3 MΩ) filled with 3 M KCl solution. Oocytes were placed in a recording chamber and perfused at a flow rate of 10 to 12 ml/min with Ringer's solution, pH 7.4, containing (in mM): 115 NaCl, 5 NaOH, 2.5 KCl, 1.8 CaCl_2_, and 10 HEPES. Membrane currents (d.c., 1 kHz) were recorded using a Warner OC-725C amplifier (Warner Instrument, Hamden, CT) and digitized at 500 Hz. Agonists were dissolved in the perfusion solution and applied using a computer-driven valve system. All recordings consisted of successive applications of α,β-meATP (10 μM) 4 min apart. Dioctanoyl (diC_8_)-phospholipids were dissolved in PBS, and 25 nL were injected in order to reach an intracellular concentration of approximately 200 μM. Inside-out macropatch experiments were performed using an Axopatch 200B amplifier (Axon Instruments, Union City, CA). PClamp 9 was used for data acquisition and analysis. The vitelline membrane of *Xenopus *oocytes was removed using fine forceps before recordings. Electrodes with resistance of 0.5–1.0 MΩ were used. The currents were evoked by ramps from -100 mV to +100 mV. The time-course of the currents was analyzed at -80 mV. DiC_8_-PIP_2 _was dissolved in the bath solution. The agonist (20 μM ATP) was applied through a gravity-driven perfusion system. For each experiment, a minimum of two batches of oocytes were tested.

### Lipid binding assay

Oligonucleotides coding for the C-terminus (F_346_-T_364_, L_344_-F_358_, and F_346_-H_397_) or the N-terminus (D_5_-I_23_) of P2X3, or the P2X2 C-terminus (F_355_-T_372_), were inserted into the pGEX-2T vector (New England Biolabs). Induced GST fusion proteins were isolated on glutathione-sepharose beads. After 4 washing cycles with PBS, the GST-proteins were eluted with 50 mM Tris-Cl (pH 8) + 5 mM reduced glutathione, and analysed by SDS-PAGE. The lipid binding analysis of the recombinant GST-fusion proteins was conducted using lipid coated hydrophobic membranes (PIP Strips™, Echelon Biosciences Inc.). The membranes were first blocked with TBS+T solution supplemented with 3% BSA for one hour at room temperature, then incubated overnight in TBS+T with 3% BSA and 1 μg/mL of the GST fusion protein. The membranes were then washed with TBS+T six times, 5 minute per wash. The primary antibody (mouse anti-GST, 1:1000) was then added in TBS+T, 3% BSA solution for one hour. Washes with TBS+T were repeated, followed by incubation with the secondary antibody (goat anti-mouse HRP, 1:5000) in TBS+T, 3% BSA. The membranes were washed again in TBS+T, and then bound proteins were detected in ECL (PerkinElmer).

### Biotinylation of surface proteins and Western blotting

Transfected HEK293 cells were incubated with Sulfo-NHS-LC-Biotin (0.5 mg/mL, Pierce). Cells were homogenized and 100 mg of proteins was adsorbed on immobilized streptavidin. Intracellular proteins were collected from the unbound fraction. Biotinylated proteins were eluted from the streptavidin beads by boiling, loaded onto a 10% SDS-PAGE then transferred to a nitrocellulose membrane. Monoclonal anti-FLAG antibody (1:5000, Sigma-Aldrich) was used to detect FLAG-tagged P2X3 subunits in ECL (GE Healthcare).

### Statistical analysis

Data are presented as mean ± S.E.M. All statistical analyses for the difference in means were carried out using unpaired as well as paired Student's *t *test and one-way ANOVA followed by Bonferroni's multiple comparison tests. Normalized data were analyzed using nonparametric Mann-Whitney U test.

## Results

### Depletion of PIP_2 _decreases P2X3 current density in DRG neurons

**Figure 1:**
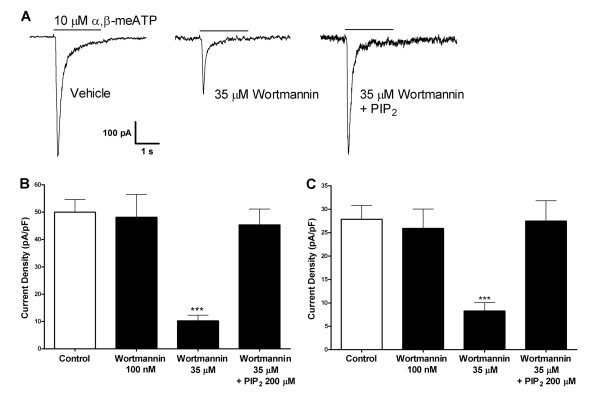
**Sensitivity of native P2X3 receptor activity to PIP_2 _depletion in DRG neurons**. A) Typical traces of P2X3 response to 10 μM α,β-meATP in DRG nociceptors (*left*), to 10 μM α,β-meATP after 2 h incubation with wortmannin (middle), to 10 μM α,β-meATP after 2 h incubation with wortmannin and intracellular application of 200 μM PIP_2 _(*right*). B) Quantitative results. P2X3 responses to 10 μM α,β-meATP under control conditions, after 2 h incubation with 100 nM wortmannin, 35 μM wortmannin, or 35 μM wortmannin with 200 μM PIP_2 _in the pipette solution (N = 6–13). C) Quantitative results. P2X3 responses to 10 μM ATP under control conditions, after 2 h incubation with 100 nM wortmannin, 35 μM wortmannin, or 35 μM wortmannin with 200 μM PIP_2 _with in the pipette solution (N = 7–18). (***, *P *< 0.001).

We investigated the role phosphoinositides might have on native P2X3 receptor currents by disrupting the synthesis of PIP_3 _and/or PIP_2_with wortmannin in cultured DRG neurons. Wortmannin, a furanosteroid metabolite of the fungi *Penicillium funiculosum*, has been extensively used to block key lipid kinases in the phosphoinositides synthesis pathways. It is a potent inhibitor of PI3K at nanomolar concentrations while it also blocks PI4K at micromolar concentrations [27,28]. In agreement with Petruska and coll. [29], small diameter neurons dissociated from adult rat DRG responded to 10 μM α,β-meATP (Fig. 1A), or 10 μM ATP, with an inward current that rapidly peaked and decayed to baseline, indicating complete desensitization. Incubation for 2 h with 35 μM wortmannin caused a 77% decrease in endogenous P2X3 current activity in response to 10 μM α,β-meATP compared to vehicle treatment (Fig. 1A, B) without affecting recovery (data not shown). P2X3 activity under control conditions had a current density of 50.0 ± 4.6 pA/pF, while P2X3 activity after wortmannin incubation had a current density of only 10.2 ± 2.1 pA/pF (Fig. 1B and Table 1). Treatment of DRG neurons with 35 μM wortmannin did not have any impact on cell capacitance (vehicle-treated: 28.19 ± 0.93 pF; wortmannin-treated: 26.61 ± 1.82 pF, p = 0.44, N = 34–86), excluding a non-specific effect of wortmannin on membrane internalization. Lowering the concentration of wortmannin to 100 nM, whereby it selectively inhibits PI3K, caused no significant changes in P2X3 activity compared to control. P2X3 current density following 2 h incubation with 100 nM wortmannin was 48.2 ± 8.3 pA/pF (Fig. 1B).

**Table 1:** Differential effects of 35 μM wortmannin treatment on P2X3 current amplitude (%) in DRG neurons and recombinant expression systems.

	Control	Wortmannin
*DRG neurons*	100.0 ± 9.3	20.4 ± 4.2 ***
*HEK293 cells*	100.0 ± 9.2	107.6 ± 21.0
*Xenopus oocytes*	92.8 ± 6.3	94.2 ± 12.2

*** *P *< 0.001

### Intracellular PIP_2 _rescues native P2X3 current responses after wortmannin treatment

To examine the direct involvement of phosphoinositides in the modulation of P2X3 current responses by wortmannin, we carried out experiments in which diC8-PIP_2 _was added to the patch pipette solution to reach intracellular concentrations of 200 μM. Notably, no modulatory effect of diC8-PIP_2 _was observed on baseline activity of P2X3 channels (data not shown), suggesting that endogenous PIP_2 _is saturating under basal conditions. Nonetheless, the inhibitory effect on P2X3 channel responses in conditions of wortmannin-induced depletion was reversed by intracellular application of the exogenous PIP_2 _analogue, as illustrated in Fig. 1B (control: 50.0 ± 4.6 pA/pF; wortmannin + diC8- PIP_2_: 45.4 ± 5.7 pA/pF). Since under normal and pathological conditions, activation of native P2X receptors is triggered by endogenous ATP, the above described experiment was also carried out using ATP as agonist (Fig. 1C). As expected, P2X3 current responses to one single application of 10 μM ATP demonstrated the same sensitivity to both wortmannin and PIP_2_, as the ones recorded with 10 μM α,β-meATP (control: 27.9 ± 3.0 pA/pF; wortmannin: 8.3 ± 1.8 pA/pF; wortmannin + diC8-PIP_2_: 27.5 ± 4.4; Fig. 1C).

### Depletion of phosphoinositides decreases P2X2/3 current density in DRG neurons

**Figure 2:**
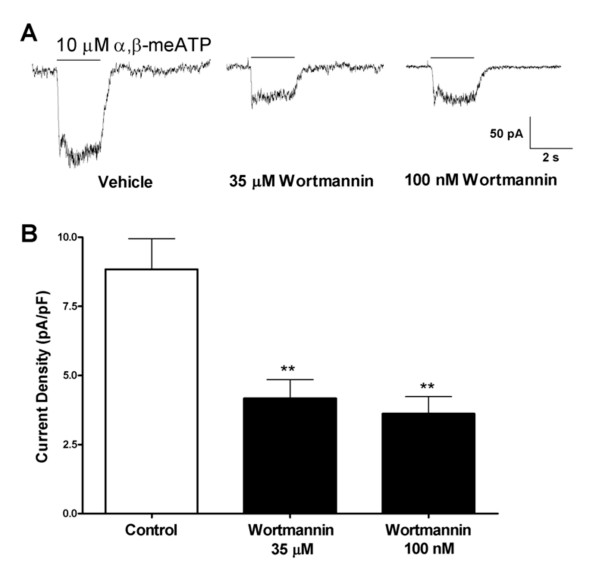
**Sensitivity of native P2X2/3 receptor activity to PIP_3 _depletion in DRG neurons. **A) Sample traces demonstrating the effects of 35 μM (*middle*) and 100 nM wortmannin incubation (2 h) (*right*) on P2X2/3 response to 10 μM α,β-meATP in DRG (control on *left*). B) Pooled data of P2X2/3 responses to 10 μM α,β-meATP under control condition, after 2 h incubation with 35 μM or 100 nM wortmannin (N = 5–6). (**, *P *< 0.01)

Although P2X3 is the predominant P2X receptor subunit expressed in DRG nociceptors, about one over ten neurons responded with a rapidly-activating and slowly-desensitizing response (Fig. 2A), attributed to the activation of heteromeric P2X2/3 receptor channels [30]. Contrary to homomeric P2X3 channels expressed in DRG neurons, α,β-meATP-evoked responses of native heteromeric P2X2/3 channels were sensitive to both high as well as low wortmannin concentration. As shown in Fig. 2A and 2B, incubation with 35 μM and 100 nM wortmannin decreased P2X2/3 current responses by 53% and 59% of control level respectively (control: 8.8 ± 1.1 pF/pA; 35 μM wortmannin: 4.2 ± 0.6 pF/pA; 100 nM wortmannin: 3.6 ± 0.6 pF/pA, N = 5–6).

### Phosphoinositides modulate P2X2/3 receptors in heterologous expression system

**Figure 3:**
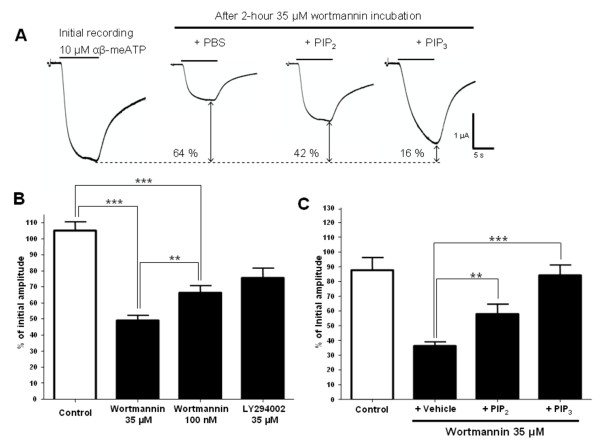
**Sensitivity of P2X2/3 currents to changes in PIP_2 _and PIP_3 _levels in *Xenopus *oocytes**. A) Sample traces showing the effect of 35 μM wortmannin incubation and the injection of PBS, PIP_2 _or PIP_3 _as compared to an initial current recorded prior to incubation. B) Pooled data of P2X2/3 responses to 2 h incubation in control solution (Barth's solution with DMSO), in 35 μM wortmannin, 100 nM wortmannin, or 35 μM LY294002 (N = 10–33). C) Pooled data of P2X2/3 current inhibition by 2 h incubation with 35 μM wortmannin in oocytes injected with vehicle, PIP_2_, and PIP_3 _(N = 5–17). (**, *P *< 0.01; ***, *P *< 0.001)

We also investigated the modulation of heteromeric P2X2/3 receptors by phosphoinositides by testing the effect of their depletion on α,β-meATP-evoked currents in *Xenopus *oocytes expressing rat P2X2/3 channels. Consistent with the results shown for the heteromeric P2X2/3 receptor recorded in DRG neurons, α,β-meATP-evoked currents in oocytes were sensitive to high as well as low wortmannin concentration. As shown in Fig. 3A and 3B, 2 h incubation of the oocytes in 35 μM or 100 nM wortmannin significantly decreased P2X2/3 responses by 51% and 34% of control level respectively (Table 2). The magnitude of the modulation of P2X2/3 current amplitudes induced by the two concentrations of wortmannin were also significantly different (*P *= 0.0035). We then tested the effect of 35 μM LY294002, a compound that selectively blocks PIP_3 _formation. After 2 h incubation, P2X2/3 current amplitudes were decreased by 24% compared to control. No significant difference of the decrease in P2X2/3 current amplitudes was found between the selective LY294002 and 100 nM of wortmannin. However, at 35 μM wortmannin (PIP_3 _and PIP_2 _depletion), current responses evoked by α,β-meATP were significantly decreased compared with those under LY294002 treatment (*P *= 0.0002).

**Table 2:** Inhibitory effects of wortmannin treatment on P2X2/3 current amplitude (%).

	Control	100 nM Wortmannin	35 μM Wortmannin
*DRG neurons*	100.0 ± 12.6	47.2 ± 7.6 **	40.9 ± 7.0 **
*Xenopus oocytes*	105.1 ± 5.5	66.3 ± 4.6 ***	49.1 ± 3.2 ***

** *P *< 0.01, *** *P *< 0.001

To verify if the effects of wortmannin were directly related to the levels of phospholipids in the oocyte, we conducted experiments where diC_8_-PIP_2 _or diC_8_-PIP_3 _was injected in the oocyte cytoplasm. Under basal conditions, injection of both diC_8_-PIP_2 _and diC_8_-PIP_3 _did not induce any significant change to the P2X2/3 current responses (data not shown). However, under depletion conditions with 35 μM wortmannin, diC_8_-PIP_2 _injected oocytes showed 42% decrease in P2X2/3 current amplitudes, compared to the 64% decrease in sham (PBS) injected oocytes (Fig. 3A, B). The involvement of PIP_3 _on the modulation of P2X2/3 receptors was also tested in the same manner, and the strong wortmannin-induced decreased response was completely rescued by the addition of diC_8_-PIP_3_. The decrease in current amplitude after diC_8_-PIP_3 _injection was 16%, a significantly smaller decrease than what was seen in sham-injected oocytes (*P *< 0.0001). Control oocytes that were injected with PBS and incubated in Barth's solution were also tested. The injection and incubation procedure induced a small non-significant decrease in P2X2/3 current amplitude (Fig. 3C).

### PIP_2 _rescues P2X3 and P2X2 current responses from rundown in excised inside-out macropatches

**Figure 4:**
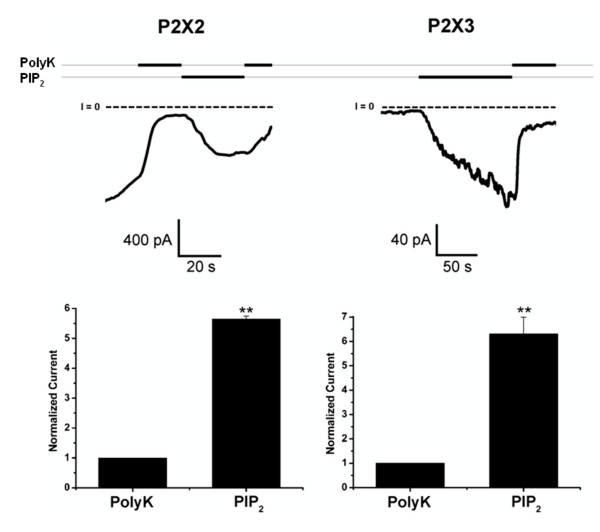
**PIP_2_-activated P2X2 and P2X3 currents in excised inside-out macropatches from *Xenopus *oocytes**. The pipette solution contained ATP at 100 μM for P2X2 and at 30 μM for P2X3. The currents were evoked by voltage ramps from -100 mV to +100 mV. The traces shown were constructed by connecting the current points at -80 mV. (**, *P *< 0.01)

Electrophysiological measurements in the inside-out macropatch configuration were also carried out on *Xenopus *oocytes expressing either P2X2 or P2X3 subunits. Once the inside-out configuration was obtained, a gradual current rundown was seen due to hydrolysis of PIP_2 _by lipid phosphatases. Polylysine was added to induce a further current rundown by binding anionic phospholipids. When PIP_2 _was then applied to the membrane patch facing the intracellular side of the membrane, it activated both P2X2 and P2X3 receptors (Fig. 4). Compared to the small residual currents seen during polylysine-induced rundown, the strong current increase evoked by PIP_2 _application was very significant for both P2X subtypes (*P *< 0.01, N = 4–9).

### No direct binding of phosphoinositides to the P2X3 subunit

**Figure 5:**
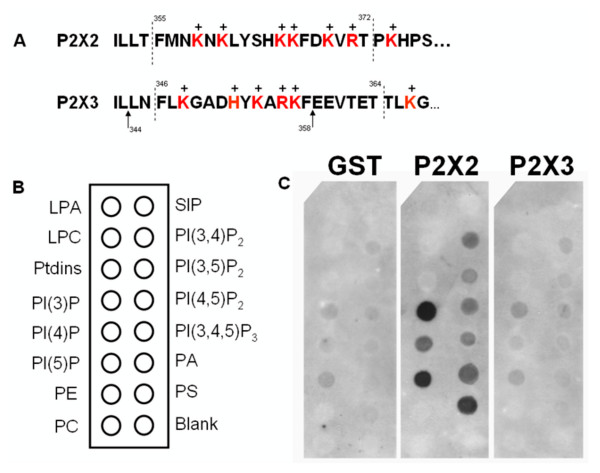
**The C-terminus of P2X2, but not of P2X3, directly binds phosphoinositides**. A) Alignment of the proximal C-termini of rat P2X2 and P2X3 subunits showing intracellular basic residues candidates for lipid binding. The dashed lines delineate the sequences of the peptides used in the lipid binding assay. B) Positions of the different lipids spotted on the membrane. C) Binding pattern of GST protein alone, GST-P2X2 (F355-T372) and GST-P2X3 (F346-T364) shows that the sequence from P2X2, but not from P2X3, directly binds several anionic phospholipids including PIP_2 _and PIP_3_.

Phosphoinositides are negatively charged lipids, therefore we investigated potential interactions with basic residues on the intracellular domain of the P2X3 channel subunit and confirmed P2X2 subunit binding using a lipid strip assay. The proximal region of the P2X3 C-terminal domain contains a cluster of basic amino acids, and was a candidate sequence for phospholipid binding. The F_355_-T_372 _P2X2 peptide as well as the F_346_-T_364 _P2X3 peptide (Fig. 5A) expressed as a GST-fusion protein were tested against a set of major phospholipids on PIP strips™ (Fig. 5B). Fig. 5C illustrates the typical binding pattern of P2X2 and P2X3 C-terminal peptides. The P2X2 peptide showed direct binding to all phosphatidylinositol phosphates: PI(3)P, PI(4)P, PI(5)P, PI(3,4)P_2_, PI(3,5)P_2_, PI(4,5)P_2_, PI(3,4,5)P_3_. It also showed strong binding to phosphatidic acid and phosphatidylserine. On the other hand, the F_346_-T_364 _P2X3 peptide did not bind to any of the lipids spotted on the membrane. A shorter peptide from the same region of the P2X3 subunit, L_344_-F_358_, and a longer peptide corresponding to the complete C-terminus, F_346_-H_397_, were also tested and did not show any binding (data not shown). Similarly, the N-terminal peptide (D_5_-I_23_) showed no phosphoinositide affinity. Increasing the concentration of the P2X3 GST fusion proteins from 1 μg/mL to 5 μg/mL did not result in significant binding.

### Phosphoinositides modulate the rundown of P2X3 receptor currents in heterologous expression systems, which was reversed by mutation R356Q

**Figure 6:**
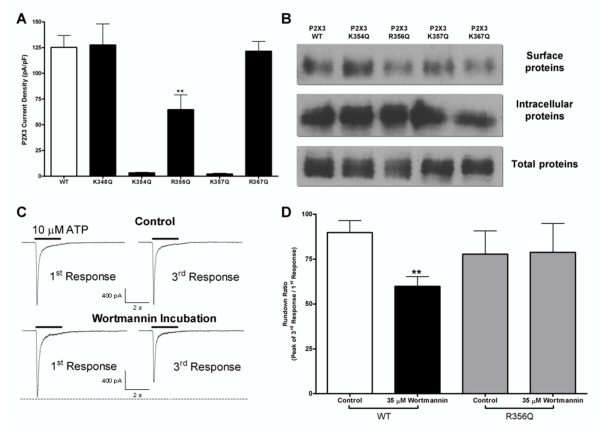
**Sensitivity of P2X3 current to PIP_2 _depletion in HEK293 cells is reversed by R356Q mutation**. A) Pooled data of wild-type and mutant P2X3 responses to 10 μM ATP expressed in HEK293 cells (N = 5–11). B) Representative surface biotinylation data of wild-type and mutant P2X3 receptor expression. C) Sample traces showing 35 μM wortmannin-induced rundown of wild-type P2X3 currents in HEK293 cells. D) Quantitative results. Rundown of wild-type and R356Q mutant P2X3 current in response to 35 μM wortmannin incubation (N = 5–6). (**, *P *< 0.01).

To further investigate the modulation of P2X3 receptor channels by phosphoinositides observed in DRG, mutation studies were conducted to identify residues on the P2X3 subunit that are critical for the interaction with PIP_2_. Interestingly, we observed that the initial current amplitude of recombinant P2X3 receptors expressed in HEK293 cells and *Xenopus *oocytes was not sensitive to PIP_2 _and PIP_3 _depletion by incubation with 35 μM wortmannin (Table 1). We previously reported that basic residues play a key role in modulation of several P2X receptor subtypes by phospholipids [20,25,26] so five point mutations (K348Q, K354Q, R356Q, K357Q, and R367Q) were generated to target candidate lysine and arginine residues involved in direct or indirect modulation of P2X3 by phosphoinositides. Of the five mutant P2X3 channel subunits produced, only the K348Q, R356Q and R367Q mutants responded to 10 μM α,β-meATP. K348Q and R367Q were indistinguishable from wild-type P2X3 channels while the mutants K354Q and K357Q were silent (Fig. 6A). The R356Q mutant displayed a 48% (64.7 ± 14.3 pA/pF) reduction in activity compared to wild-type P2X3 channel responses (125.2 ± 11.6 pA/pF). To check if the decreased responses of the R356Q mutant were actually due to a decreased receptor function rather than a decreased receptor trafficking, a surface biotinylation study was performed with all the mutants and the wild-type P2X3 constructs. Surface protein expression for HEK293 cells transfected with wild-type P2X3 was not significantly different from the one in HEK293 cells transfected with any of the mutants (Fig. 6B).

Using three consecutive applications of 10 μM α,β-meATP at 4 min intervals we discovered that the recovery of recombinant P2X3 receptors was sensitive to phosphoinositide depletion (Fig. 6C). Under control conditions, a 4 min washout between each agonist application allowed recombinant P2X3 receptor responses to almost fully recover (93.1 ± 4.9%, N = 7), whereas wortmannin-induced phosphoinositide depletion led to a significantly decreased recovery (62.6 ± 7.0%, N = 10; *P *= 0.0053; Fig. 6D and Table 3). The R356Q mutation reversed the sensitivity of recombinant wild-type P2X3 receptor channels to modulation by PIP_2_. Incubation with 35 μM wortmannin for 2 h produced no significant changes to the recovery rate of currents elicited by 10 μM ATP in HEK293 cells transfected with the R356Q mutant (Fig. 6D). Incubation with wortmannin produced no significant changes to the initial current amplitude of the mutant P2X3 R356Q. These results indicate a role for the cytoplasmic residue R356 in the sensitivity of P2X3 to phosphoinositides, possibly through binding to an unidentified partner protein.

**Table 3:** Differential effects of 35 μM wortmannin treatment on P2X3 current rundown (%) in DRG neurons and recombinant expression systems.

	Control	Wortmannin
*DRG neurons*	96.0 ± 7.5	92.0 ± 5.3
*HEK293 cells*	89.9 ± 6.6	59.8 ± 5.4 **
*Xenopus oocytes*	93.1 ± 4.9	62.6 ± 7.0 **

** *P *< 0.01

## Discussion

### Native P2X3 receptor function is sensitive to PIP_2 _levels

This study identified a novel regulatory role of phosphoinositides in homomeric and heteromeric P2X3-containing receptor-channels, in native and recombinant forms. Functional homomeric P2X3 and heteromeric P2X2/3 receptor channels are highly expressed on DRG primary sensory neurons that transmit nociceptive sensory information. Native P2X3 receptor currents evoked in rat DRG nociceptors by the selective P2X agonist α,β-meATP were sensitive to high concentrations of wortmannin which deplete PIP_2 _and PIP_3_, but not to low concentrations of wortmannin which deplete PIP_3 _only. The addition of PIP_2 _into the recording pipette rescued P2X3 peak currents under wortmannin-induced phosphoinositide-depleted condition, providing unequivocal evidence for the regulatory role of PIP_2 _on P2X3 receptor activity. Dialysis with PIP_2 _analog into the recording pipette did not affect P2X3 currents under basal condition, indicating saturation by endogenous PIP_2 _in DRG neurons in primary culture. Altogether, these data support the involvement of PIP_2 _over PIP_3 _on endogenous P2X3 receptor response. Furthermore, the absence of effect in basal conditions on P2X3 current responses indicates that no unwanted parallel pathways were modulated by the addition of phosphoinositides. Increases in intracellular Ca^2+ ^have also been shown to stimulate PIP_2 _synthesis via PI4K [31]. PKC was shown to directly activate PI4K [32] and to promote PIP_2 _availability [33]. However, preincubation of DRG neurons with 500 nM staurosporine, a wide-spectrum PKC blocker, did not alter P2X3 currents (data not shown), indicating there may be another staurosporine-insensitive mechanism for the saturation of PIP_2 _levels in cultured DRG neurons.

Interestingly, we could not reproduce in heterologous expression systems the effect of PIP_2 _depletion on the first current response of native P2X3 receptors. Instead, P2X3 receptors expressed in oocytes and HEK293 cells showed decreased speed of recovery of receptor responses under PIP_2 _depletion condition. Moreover, PIP_2 _was able to rescue P2X3 currents from rundown in excised patches. The simplest working hypothesis for the difference observed between recombinant and native P2X3 responses to wortmannin-induced depletion of PIP_2 _is the existence of a critical component of P2X3 receptor function expressed in DRG neurons, but absent in *Xenopus *oocytes and HEK293 cells.

### Heteromeric P2X2/3 receptor function is sensitive to PIP3 levels

The modulation of the heteromeric P2X2/3 receptors by phosphoinositides was also investigated in native DRG neurons. A subpopulation of small diameter/nociceptive DRG neurons has been shown to express α,β-meATP-evoked currents with slow onset and slowly-desensitizing responses due to the activation of heteromeric P2X2/3 receptor channels [30]. Our study shows that the effects of PIP_2 _and PIP_3 _depletion on α,β-meATP-evoked P2X2/3 current amplitudes in DRG neurons were the same in magnitude, indicating that PIP_3 _played a major role. Nonetheless, using *Xenopus *oocytes expressing P2X2/3 receptors, we observed an inhibitory effect on current amplitudes that was stronger at high than at low wortmannin concentrations, and partial with the selective PI3K inhibitor LY294002. Although PIP_3 _completely rescued tandem P2X2/3 current amplitudes under wortmannin-depleting condition, PIP_2 _was found to have a partial albeit significant effect. This result further supports a predominant role of PIP_3_, but does not exclude PIP_2_, on the modulation of P2X2/3 receptors. Fujiwara and coll. [23] reported that homomeric P2X2 receptor channels were sensitive to wortmannin and to the PI3K specific inhibitor LY294002, as decreased current amplitudes were recorded in *Xenopus *oocyte expression system. However, taking into consideration lipid binding to recombinant C-terminal P2X2 channel constructs, they proposed that PI(3)P and PI(3,5)P_2_, rather than PIP_2 _or PIP_3_, are the main regulators of homomeric P2X2 function. Likely due to less stringent experimental conditions, we show a significant binding of the C-terminus of P2X2 to PIP_2 _and PIP_3_, as well as many other anionic phospholipids (Fig. 5).

### Phospholipid modulation of P2X3 receptors does not involve direct binding

Several groups have reported the direct binding of the P2X receptors to phospholipid-containing membranes, using a biochemical approach in which GST-fusion proteins of various P2X C-terminal domains suspected to mediate the binding were tested on lipid strips [23,25,26]. To test if P2X3 modulation involved direct binding to phosphoinositides, GST-fusion proteins coding for the C-terminal region of P2X3 homologous to the identified phosphoinositide binding region in other P2X receptors were generated. The C-terminus was a candidate domain as it has been shown to be involved in the modulatory function of several P2X receptor channels, most notably their desensitization [23,34,35] and membrane targeting [36-38]. Surprisingly, no binding of the recombinant P2X3 constructs to any phospholipids could be detected above background. The N-terminus of the Kir channel family has been shown to interact with PIP_2_for their gating modulation [39]. This led us to test the possibility that interaction between the P2X3 receptor and PIP_2 _involved its N-terminal domain. Again, no such interaction was observed as the binding of N-terminal P2X3 constructs to phospholipid membranes was not detected. Therefore P2X3 is the only member of the P2X family, so far, that does not display direct binding to phosphoinositides using the lipid strip assay. We cannot rule out, however, the possibility that phosphoinositide binding requires the intact P2X3 receptor subunit.

Indirect modulation of ion channels by phosphoinositides is not ungrounded. For instance, Michailidis and coll. [21] have recently shown that the regulation of the NMDA receptor by PIP_2 _requires the intracellular protein α-actinin, which serves as a partner of NMDAR2 subunit facilitating phospholipid sensing. Moreover, Kim and coll. [40] have reported a novel protein called PIRT, expressed in nociceptive neurons of DRG, which binds to TRPV1 channels and several phosphoinositides, including PIP_2_, to modulate TRPV1 activity. Overall, the requirement of a specific partner protein mediating the interaction between phosphoinositides and the P2X3 subunit may explain why the modulatory effects of phosphoinositides on P2X3 receptors seen in DRG neurons was not observed in HEK293 cells. The expression of a potential lipid-binding protein linked to P2X3 subunits remains to be investigated in DRG neurons. Activation of P2X3-containing receptors has been suggested to provide a specific mechanism wherein ATP released via synaptic transmission or tissue injury elicits nociception, with potential discrete role for P2X3 and P2X2/3 receptor activation in acute and chronic pain [13]. Our study provides evidence that in nociceptors, the function of homomeric P2X3 receptors is primarily modulated by PIP_2_, whereas the function of heteromeric P2X2/3 receptors is mostly modulated by PIP_3_. P2X receptors are co-expressed with several classes of metabotropic receptors that regulate phosphoinositide levels through the activation of PI3K or phospholipase C in non-peptidergic DRG nociceptors. So it will be interesting to investigate the role of a differential modulation of P2X3-containing receptors by phosphoinositides in pathophysiological processes such as chronic inflammatory and neuropathic pain. Moreover, searching for a phospholipid-binding protein partner of P2X3 subunits may well pave the way for designing new strategies to control pathological nociceptive transmission.

## Conclusion

This study demonstrates a modulatory role of phosphoinositides on the function of native P2X3 and P2X2/3 purinergic receptors. While the homomeric P2X3 receptor is sensitive to changes in PIP_2 _levels only, the P2X2/3 heteromeric receptor is sensitive to changes in both PIP_2 _and PIP_3 _levels. Unlike other ionotropic purinoceptors, including P2X2, which bind directly to phosphoinositides, our findings suggest that P2X3 is modulated by PIP_2 _indirectly. Understanding the mechanisms of lipid sensing that are selective for the P2X3 and P2X2/3 ATP receptor subtypes in DRG sensory neurons would aid in elucidating the respective physiological role of these prominent targets in pain therapeutics.

## Competing interests

The authors declare that they have no competing interests.

## Authors' contributions

GM, LPB, QZ, and AJCD designed and performed the experiments. GM, LPB, ARA, CQC, DL and PS participated in the manuscript writing. ARA, CQC, DL, and PS provided guidance on experimental design and data interpretation, and PS supervised the progress of the study. All the authors have read and approved the final manuscript.
